# Supplementary material for: Detection of methylation, acetylation and glycosylation of protein residues by monitoring 13C chemical-shift changes: A quantum-chemical study
Source: PeerJ. 2016 Jul 21;4:e2253. doi: 10.7717/peerj.2253 (PMC4963218; doi:10.7717/peerj.2253)
Supplement: Supplemental Information 1 — The information provided in the Supplemental Information includes: (i) the Kernel Density Estimation of the D values for Lys and Arg (Figs. S1 and S2); (ii) the probability profile distribution to detect glycosylation of Ser, Thr and Asn (Figs. S3–S5), and (iii) the ball and stick representation of a glycan-amino acidic residue for Thr and Asn (Figs. S6 and S7). [file peerj-04-2253-s001.pdf]

# Supplemental Information for

# Detection of methylation, acetylation and glycosylation of protein residues by monitoring $^{13}C$ chemical-shift changes: A quantum-chemical study

Pablo G. Garay,[1] Osvaldo A. Martin,[1] Harold A. Scheraga[2] and Jorge A. Vila[1,§]

[1]*IMASL-CONICET, Universidad Nacional de San Luis, Italia 1556, 5700-San Luis, Argentina;*

[2]*Baker Laboratory of Chemistry, Cornell University, Ithaca, NY, USA.*

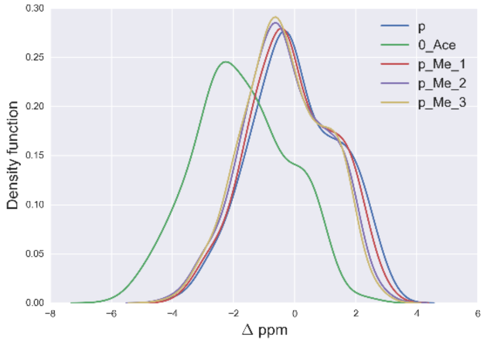


(a)

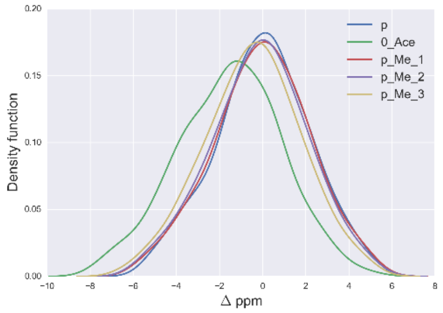


(b)

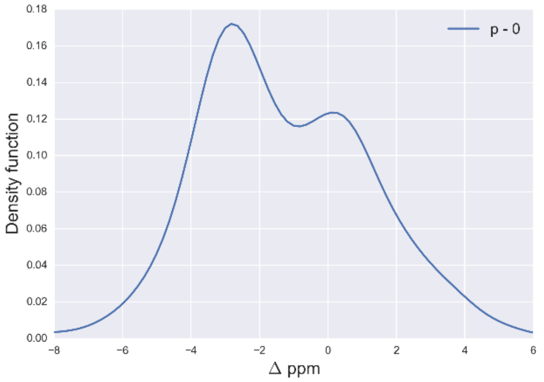


(c)

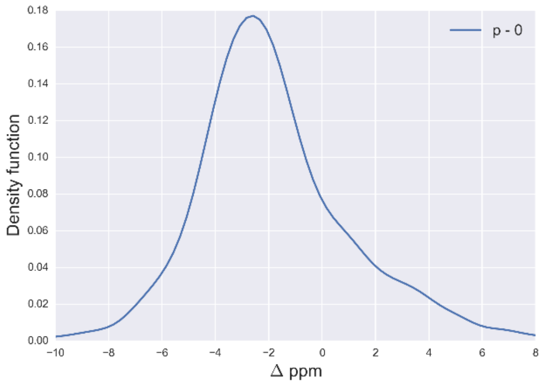


(d)

**Figure S1**.- (**a**) Kernel Density Estimation of the Δ values of the $^{13}C^{\alpha}$ nucleus of charged non-modified (blue-line), acetylated (green-line), *mono*- (red-line), *di*- (violet-line), and *tri*-methylated (yellow-line) Lys; (**b**) same as (**a**) for the $^{13}C^{\beta}$ nucleus; (**c**) Kernel Density Estimation of the Δ values of the $^{13}C^{\alpha}$ nucleus of non-modified Lys upon protonation/deprotonation; (**d**) same as (**c**) for the $^{13}C^{\beta}$ nucleus.

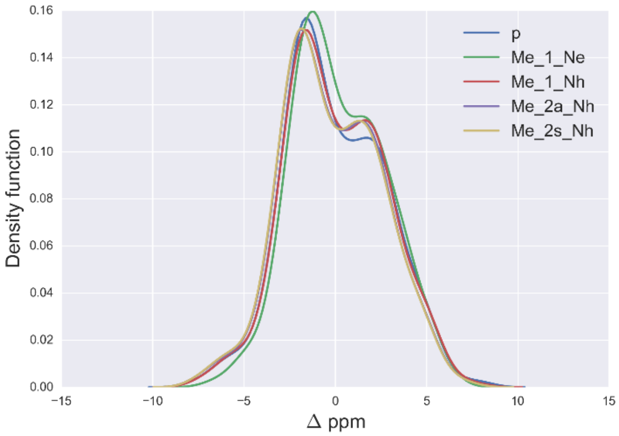


(a)

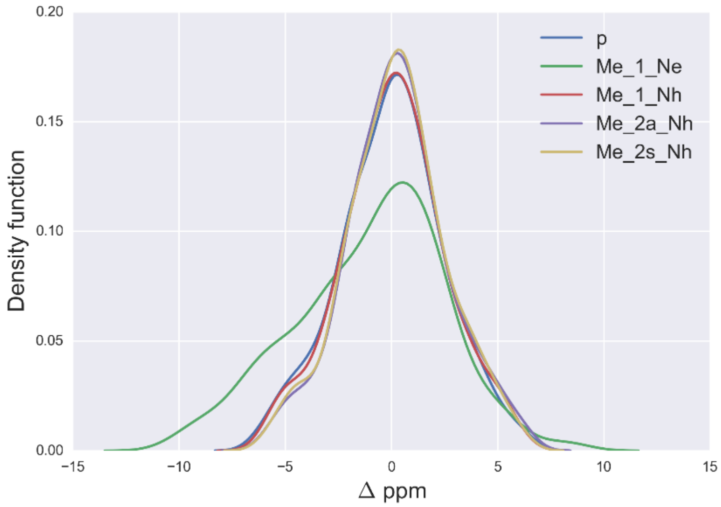


(b)

**Figure S2**.- (**a**) Kernel Density Estimation of the Δ values of the $^{13}C^{\alpha}$ nucleus of *non*-modified (blue-line), $N^{\varepsilon}$ (green-line) and $N^{\eta}$ (red-line) *mono*-methylated, asymmetric (violet-line) and symmetric (yellow-line) *di*-methylated Arg; (**b**) same as (**a**) for the $^{13}C^{\beta}$ nucleus.

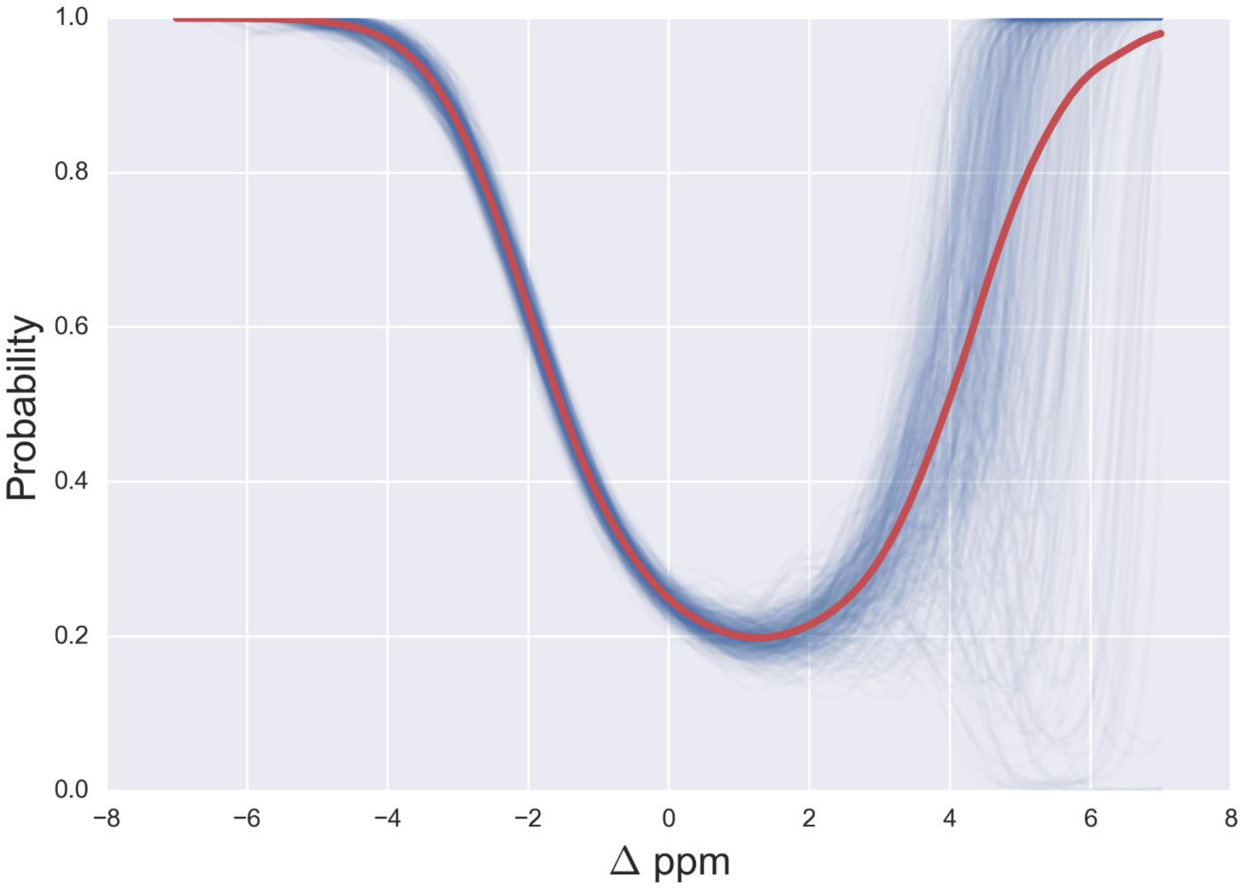


**Figure S3**. Probability to detect glycosylation of Ser, i.e., either α-D-GalpNAc-(1-O)-Ser or β-D-GlcpNAc-(1-O)-Ser, as a function of the Δ values of the $^{13}C^{\beta}$ nucleus of Ser (shown in Figure 4 in the main text). The red line represents the expected probability-profile and the blue lines the uncertainty in the data according to the Bayesian model.

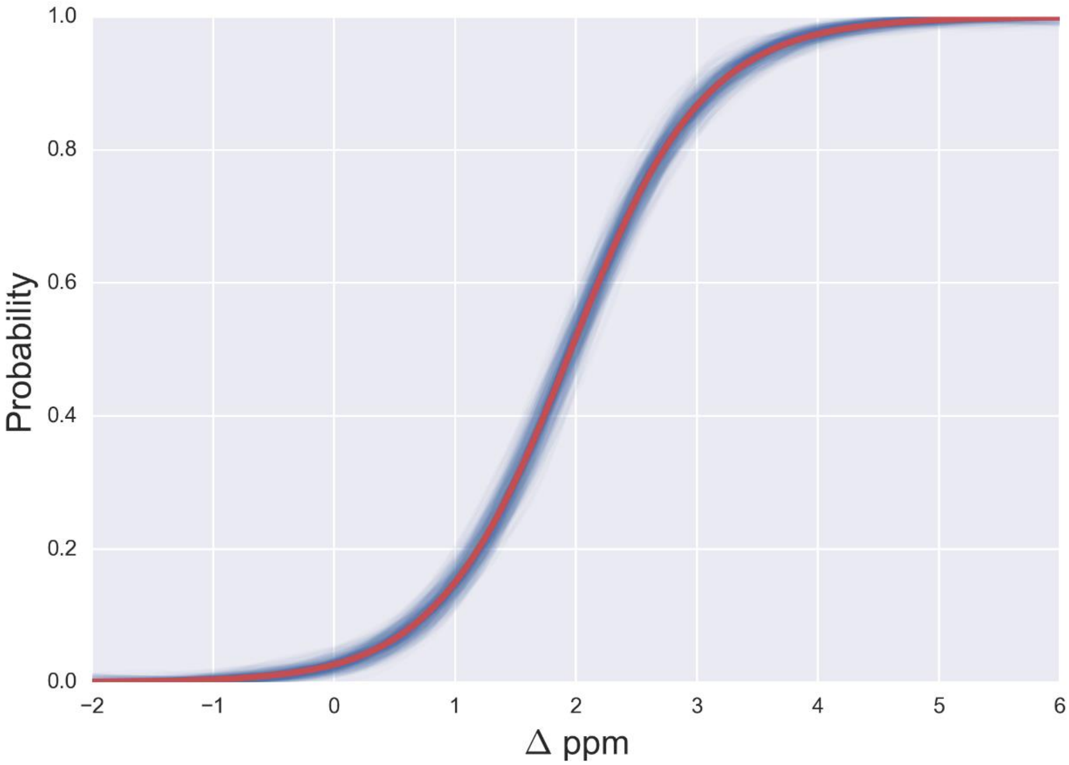


**Figure S4**. Probability to detect glycosylation of Thr [α-D-GalpNAc-(1-O)-Thr], as a function of the chemical-shift differences (Δ) for the $^{13}C^{\beta}$ nucleus of Thr (shown in Figure 6 in the main text). The red line represents the expected probability-profile and the blue lines the uncertainty in the data according to the Bayesian model.

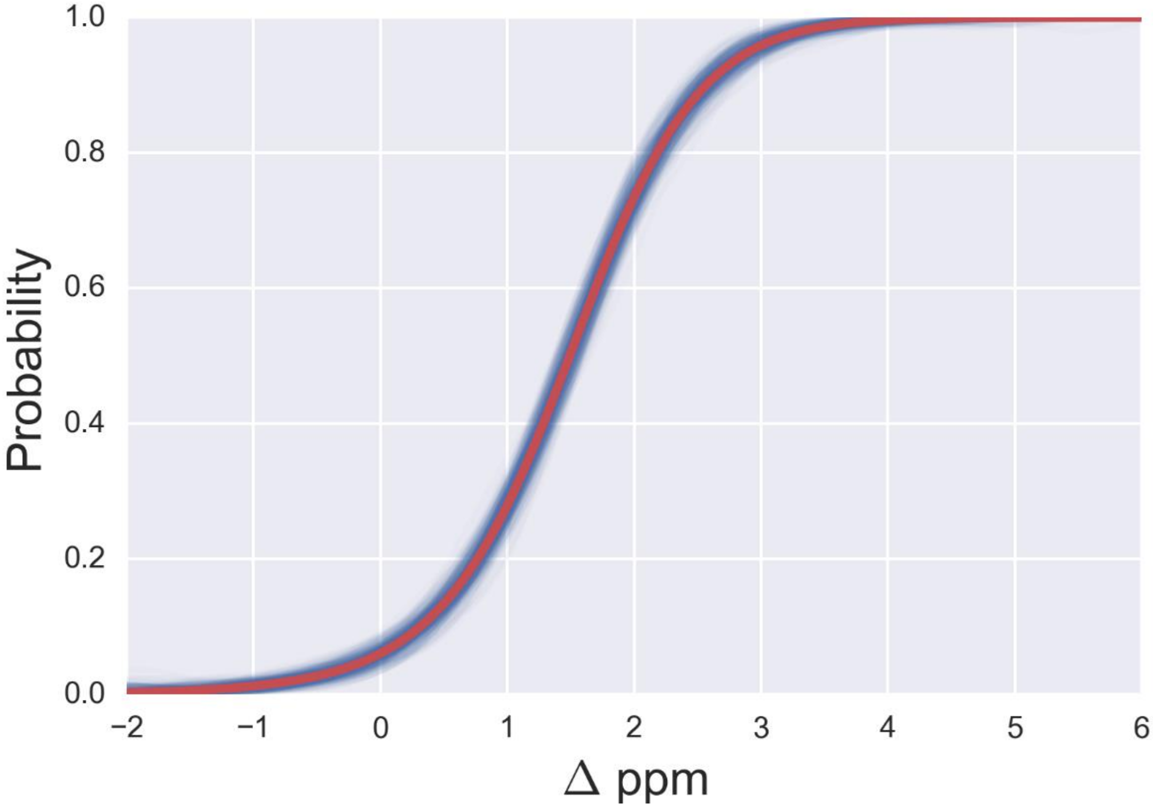


**Figure S5**.- Probability to detect glycosylation of Asn [β-D-GlcpNAc-(1-N)-Asn], as a function of the chemical-shift differences (Δ) for the $^{13}C^{\gamma}$ nucleus of Asn (shown in Figure 7 in the main text). The red line represents the expected probability-profile and the blue lines the uncertainty in the data according to the Bayesian model.

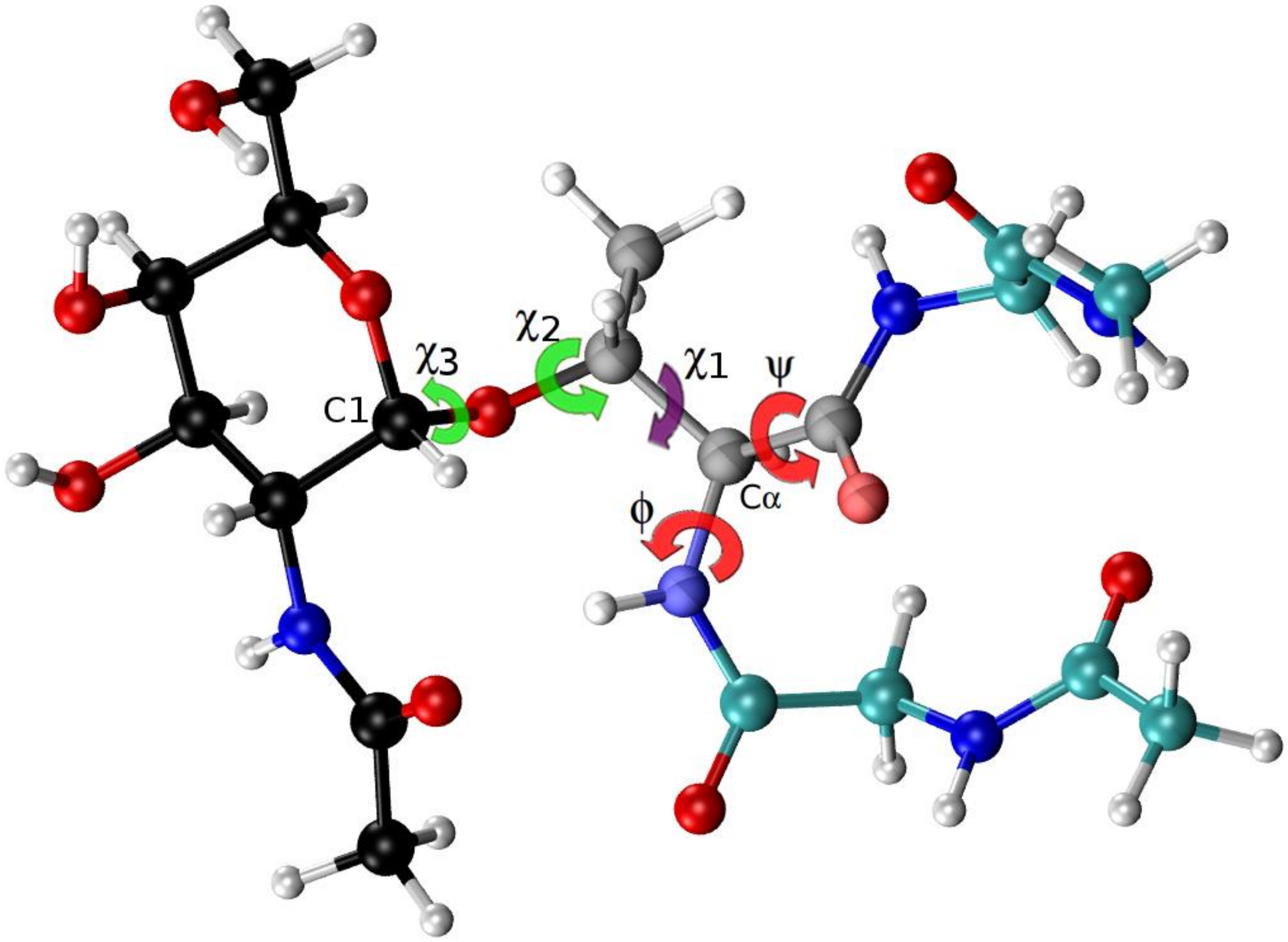


**Figure S6**.- Ball and stick representation of a glycan-amino acidic residue, namely for α-D-GalpNAc-(1-O)-Thr with "1" representing **C1** of the glycan and "O" representing the oxygen of the side-chain of Thr in an Ac-Gly-Thr-Gly-Nme tripeptide, in an arbitrary conformation. The χ2 and χ3 torsional angle, for the carbohydrate group (α-D-GalpNAc), are highlighted in green, while the one corresponding to the amino-acidic residue (Thr) are in red, for ϕ,ψ, and purple, for χ1.

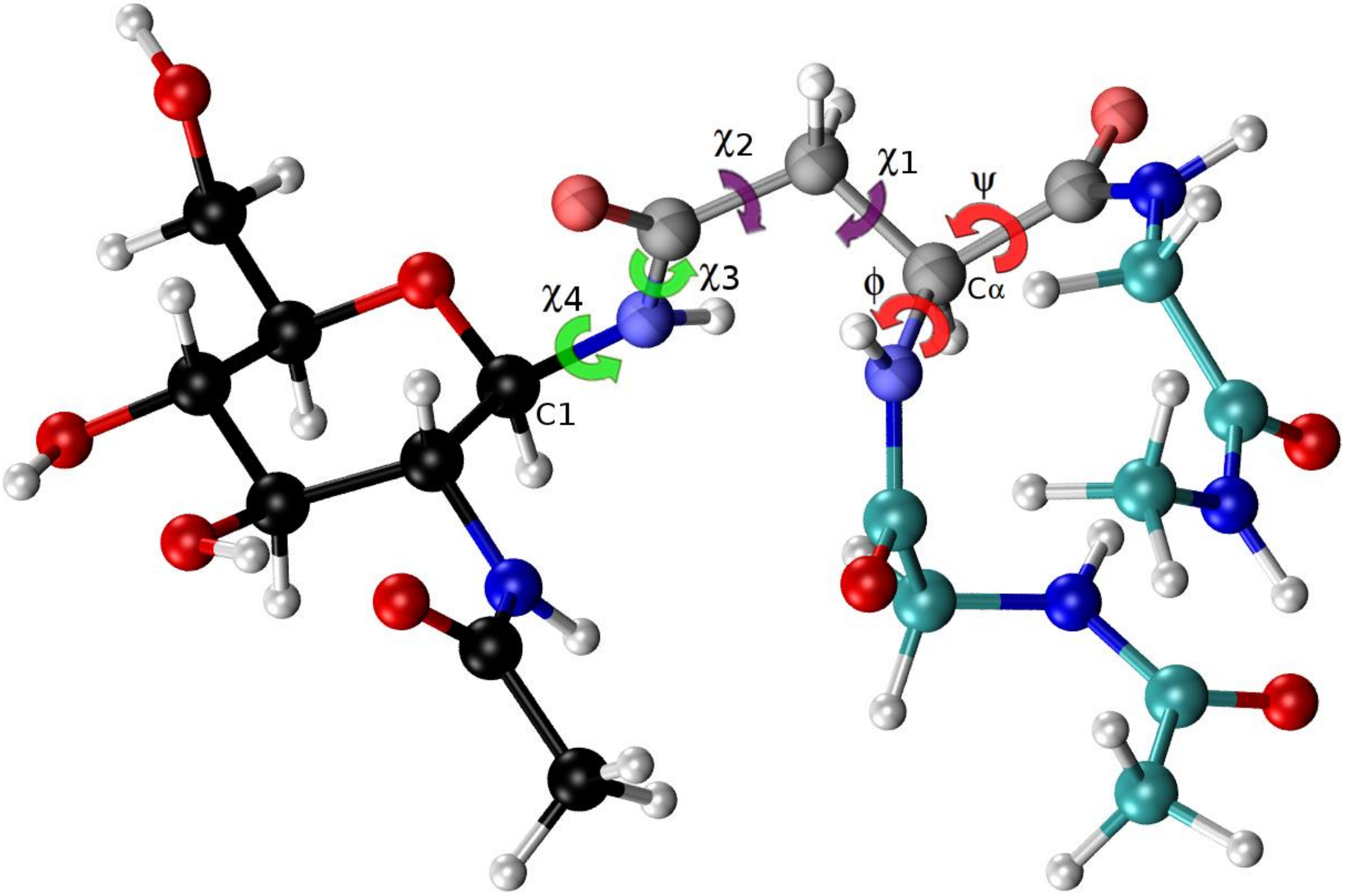


**Figure S7**.- Ball and stick representation of a glycan-amino acidic residue, namely for β-D-GlcpNAc-(1-N)-Asn with "1" representing **C1** of the glycan and "N" representing the nitrogen of the side-chain of Asn in an Ac-Gly-Asn-Gly-Nme tripeptide, in an arbitrary conformation. The χ3 and χ4 torsional angles, for the carbohydrate group (β-D-GalpNAc), are highlighted in green, while the corresponding one for the amino-acidic residue (Asn) are highlighted in red, for ϕ,ψ, and purple, for χ1 and χ2.

.
